# The path to approvals: Facilitating access to healthcare data via a local Governance Forum.

**DOI:** 10.23889/ijpds.v7i3.1981

**Published:** 2022-08-25

**Authors:** Joanne Lumsden, Katie Wilde, Sharon Gordon, Corri Black, Shantini Paranjothy

**Affiliations:** 1 University of Aberdeen

## Objectives

Research using unconsented healthcare data has the potential to have significant public benefit in terms of improved health outcomes. However, due to the sensitive nature of this data, appropriate governance, approvals, and mitigations are crucial to ensure that patient confidentiality and public trust are maintained.

## Approach

As the permissions process can be a barrier to research, we established a local Governance Forum with the aim to streamline processes, increase transparency, and document approval requirements. Governance Forum membership includes representatives from the relevant health board and university departments (e.g., the regional Safe Haven, Information Governance, Ethics Committee, Data Protection, Research and Development). To achieve the Forum’s objectives, the regional Safe Haven raises current and potential project scenarios at quarterly meetings to confirm requirements and help develop a clear understanding of the approval pathway, with specific topics discussed with the relevant members out with these meetings as required.

## Results

The Forum has strengthened communication and collaboration with Forum members which has enabled the Safe Haven to better support researchers. Outputs have included a permission pathway guidance document for researchers. This initial guidance document outlines approval requirements and provides relevant advice and support, with work currently ongoing to build on and improve this. In addition, as channels have been established for onboarding new project types and novel datasets, the diversity of datasets available for research is increasing and the route to approvals is now clearer for both researchers and internal teams. The Governance Forum activity has the potential to facilitate future research which may lead to academic publications and research funding, as well as improved health outcomes and policy change in the longer-term.

## Conclusion

Although the progress so far has had significant impacts, the approval processes remain challenging for certain projects. Therefore, it will be imperative that sufficient resources are available in the future to ensure that further progress can be made via the Governance Forum as well as national initiatives.

